# Thymidylate synthase disruption to limit cell proliferation in cell therapies

**DOI:** 10.1016/j.ymthe.2024.06.014

**Published:** 2024-06-12

**Authors:** Rocio Sartori-Maldonado, Hossam Montaser, Inkeri Soppa, Solja Eurola, Juhana Juutila, Melanie Balaz, Henri Puttonen, Timo Otonkoski, Jonna Saarimäki-Vire, Kirmo Wartiovaara

**Affiliations:** 1 Stem Cells and Metabolism Research Program, Faculty of Medicine, University of Helsinki, 00290 Helsinki, Finland; 2 Faculty of Biological and Environmental Sciences University of Helsinki, 00790 Helsinki, Finland; 3 Institute of Biotechnology, Helsinki Institute of Life Science, University of Helsinki, 00790 Helsinki, Finland; 4 Children’s Hospital, University of Helsinki and Helsinki University Hospital, 00290 Helsinki, Finland; 5 Clinical Genetics, Helsinki University Hospital, 00290 Helsinki, Finland; 6 Department of Pathology, Helsinki University Hospital, 00290 Helsinki, Finland

**Keywords:** TYMS, DNA synthesis, proliferation, safety switch, cell therapy, human pluripotent stem cells, diabetes, beta-cell differentiation

## Abstract

Stem and progenitor cells hold great promise for regenerative medicine and gene therapy approaches. However, transplantation of living cells entails a fundamental risk of unwanted growth, potentially exacerbated by CRISPR-Cas9 or other genetic manipulations. Here, we describe a safety system to control cell proliferation while allowing robust and efficient cell manufacture, without any added genetic elements. Inactivating TYMS, a key nucleotide metabolism enzyme, in several cell lines resulted in cells that proliferate only when supplemented with exogenous thymidine. Under supplementation, *TYMS*^−/−^-pluripotent stem cells proliferate, produce teratomas, and successfully differentiate into potentially therapeutic cell types such as pancreatic β cells. Our results suggest that supplementation with exogenous thymidine affects stem cell proliferation, but not the function of stem cell-derived cells. After differentiation, postmitotic cells do not require thymidine *in vitro* or *in vivo*, as shown by the production of functional human insulin in mice up to 5 months after implantation of stem cell-derived pancreatic tissue.

## Graphical abstract

**Figure:**
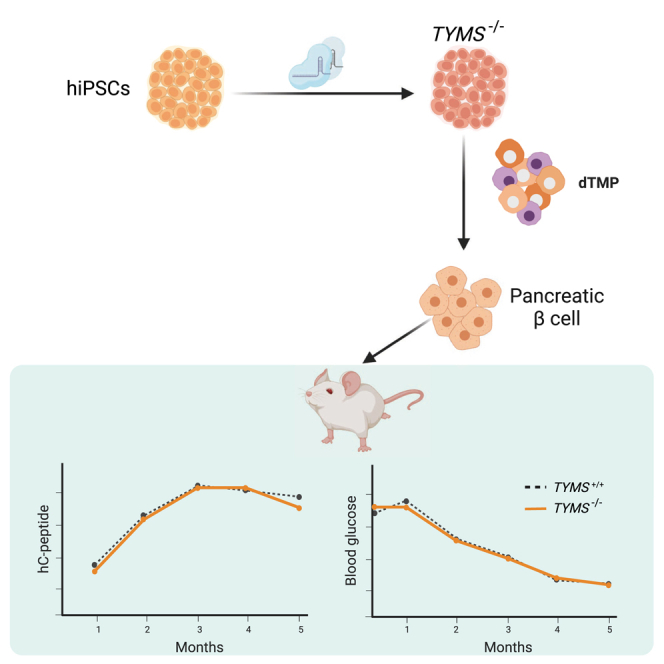

## Teaser

A genetic modification in stem cells makes their proliferation possible only under thymidine supplementation. This method is proposed to reduce the risk of uncontrolled cell proliferation for stem cell-based therapies.

## Introduction

Cell therapies offer new possibilities for previously untreatable or challenging medical conditions. Among the cell types used in such therapies, human induced pluripotent stem cells (hiPSCs) have garnered significant attention due to their availability from patient-specific somatic cells^1^^,^^2^^,^^3^^,^^4^ and their capability to differentiate into potentially therapeutic cell lineages, such as glucose-responsive insulin-secreting pancreatic islets.^5^^,^^6^^,^^7^^,^^8^ hiPSCs circumvent the ethical concerns associated with the use of embryonal stem cells,^9^^,^^10^ and, unlike donor-derived mesenchymal stem cells, their autologous source diminishes the risk of immune rejection and graft-versus-host disease.^11^^,^^12^ Furthermore, the available technologies for iPSC gene transfer and genome editing enable the development of genetically modified cell-based therapies with enhanced therapeutic potential.^13^^,^^14^

Stem cells’ flexibility and tolerance for modifications have broadened their applications to include, for example, roles as medical delivery vehicles.^15^ They further permit silencing or modification of genes involved in, for example, immune recognition.^16^ These features may enhance their compatibility for allogeneic cell therapies, reducing the need for immunosuppression compared to other cell sources. However, although the universal off-the-shelf cell products decrease the risk for immune rejection, the diminished immunogenicity raises concerns related to immune evasion and potential oncogenic events.^17^

On top of creating biologically useful cells for a desired clinical purpose, a successful biotechnological therapeutic product needs to meet the criteria for safety and scalable manufacturing. These two goals, however, often compromise each other, since manufacturing benefits from robust cell growth, but intensive or unlimited proliferation poses a threat when the cells are transferred to patients.^18^^,^^19^

Attempts to create safer cells for therapy have yielded different strategies.^20^^,^^21^ These safety systems aim to prevent uncontrolled proliferation or selectively eliminate the transplanted cells if necessary.^22^^,^^23^ Some of the existing safety strategies include the use of inducible apoptotic systems, suicide genes, antibody-mediated cell depletion, and gene editing-based switches (Table 1). Often, these switches work by reversibly activating or inactivating genes upon exposure to a small molecule (on- and off-switches, respectively). However, they all present remarkable challenges: susceptibility to genetic silencing or alteration, transgenic or viral-derived origin, slow activation speed, rapid remission, and/or potential neurotoxicity.^21^^,^^24^^,^^25^

**Table 1:** Summary table of selected “safety” systems, and potential advantages and disadvantages

Type	Function	Examples	Advantages	Disadvantages
Enzyme/prodrug^25^^,^^46^^,^^47^^,^^48^^,^^49^	Suicide transgenes into cells that transform prodrug into toxic metabolite	HSV-TK + ganciclovir^50^^,^^51^	Some have the ability to eliminate the whole graft Could be combined with other strategies Safe Generally efficient	Transgenes derive from viruses, bacteria or yeast Immuno-rejection of the therapeutic cells Toxicity of the prodrug Bystander effect Slow activation time Silencing of transgene
		CD + 5-FC^52^		
		NTR + CB1954^53^		
		PNP + MEP^54^		
		mTMPK + AZT^55^		
Monoclonal antibody mediated^49^^,^^56^	Engineer a gene targeted by an antibody into the transplanted cells	CD20t + rituximab	Avoid graft-versus-host disease^57^ Efficient Different payloads for ADC	CD20 present in endogenous B cells
		hEGFRt + cetuximab ^58^		
		c-myc tag + anti-c-myc tag^59^		
		X + ADC anti-X^60^		
Inducible dimerization^49^	Insertion of a modified caspase-9 The recruitment sequence is replaced by a dimerization domain of FK506 binding protein	iCas9^61^ + CID	Low immunogenicity Inert small molecule Fast action Tested to eliminate iPSCs^62^	Silencing of transgene CID resistance^63^
Metabolic	Endogenous disruption of pyrimidine *de novo* synthesis	*UMPS* knockout^20^	Transgene free	May affect RNA synthesis

ADC, antibody-drug conjugate; AZT, azidothymidine; CD, cytosine deaminase; CID, chemical inducer of dimerization; EGFR, epidermal growth factor receptor; HSV-TK, herpes simplex virus thymidine kinase; mTMPK, mutated thymidylate monophosphate kinase; NTR, nitroreductase; PNP, purine nucleoside phosphorylase.

**Figure 1:**
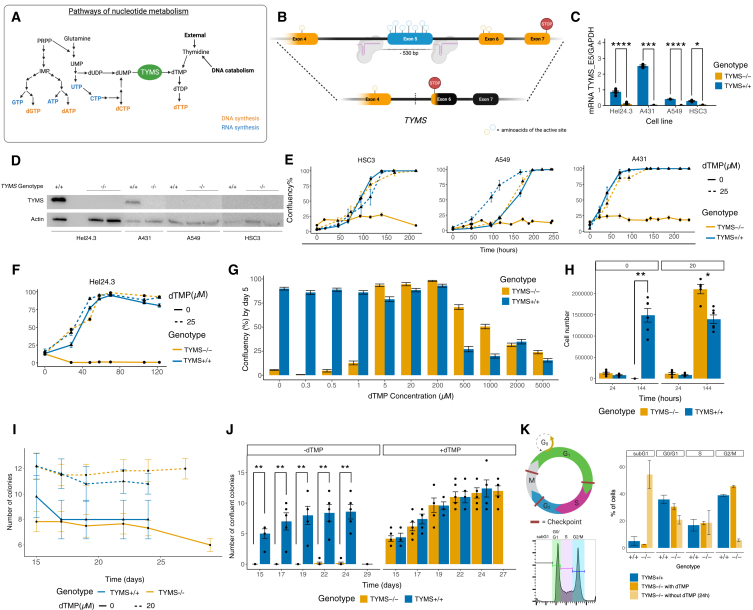
Disruption of *TYMS* makes proliferative cells dependent on thymidine supplementation (A) Diagram showing the nucleotide pathways for DNA (orange) or RNA (blue) synthesis. (B) Schematic representation of the gene of interest (*TYMS*), showing the 2 gRNAs, and the amino acids that form the active site of the protein over the corresponding exon that code for them. The lower line represents the change of frame upon DNA repair. (C) *TYMS* expression in wild-type versus knockout hiPSC (HEL24.3), A431, A549, and HSC3 (*n* = 3/cell line). Results shown as ratio of *TYMS* mRNA (primer targeting exon 5) over glyceraldehyde 3-phosphate dehydrogenase (GAPDH) mRNA contents. (D) Western blot showing the absence of *TYMS* upon inactivation of its gene. (E) Confluency-based growth curve of wild-type and knockout cancer cells supplemented with 0 or 25 μM dTMP. Results shown as average confluency ± SE. (F) Confluency-based growth curve of wild-type and knockout iPSCs supplemented with 0 or 25 μM dTMP. Results shown as average confluency ±SE. (G) Confluency at day 5 of wild-type and knockout hiPSCs under different concentrations of dTMP (0–5000 μM). Results shown as average confluency per image ± SE. (H) Cell count of wild-type and knockout iPSCs supplemented with 0 or 20 μM dTMP at 24 and 144 h postseeding. (I) Number of wild-type and knockout monoclonal colonies (N/48) supplemented with 0 or 20 μM dTMP. (J) Number of wild-type and knockout monoclonal colonies supplemented with 0 or 20 μM dTMP that reached over 90% confluency. (K) Summary of flow cytometry results for cell-cycle analysis. Statistical significance in (C), (H), and (J) based on Wilcoxon test; ns (not shown) *p* > 0.05; ∗*p* < 0.05; ∗∗*p* < 0.01; ∗∗∗*p* < 0.001; ∗∗∗∗*p* < 0.0001. See also Figure S1.

Here, we envisioned an endogenous safety strategy by disrupting a key metabolic gene, whose function can be compensated by administering an easily available compound. Using CRISPR-Cas9, we genetically inactivated thymidylate synthase (*TYMS*), the only known enzyme in charge of the *de novo* thymidylate (dTMP) synthesis.^26^ This knockout generates a rescuable auxotrophy to thymidine and restricts the synthesis of DNA, without altering normal RNA or protein production (Figure 1A). This means that dividing cells depend on the external supplementation of thymidine to proliferate and achieve robust manufacturing of the cells of interest. However, once the cells have exited the cell cycle and terminally differentiated, they do not require supplementation to synthesize nucleic acids. While preventing dividing cells from proliferating uncontrollably, this approach does not require the use of small molecules or the insertion of large transgenic elements. Hence, it cannot be silenced by mutagenesis, and it does not result in leakiness or immunogenicity.

Our results provide evidence that the culture and proliferation of *TYMS*^−/−^ cells can be regulated with exogenous dTMP *in vitro* and *in vivo*. Furthermore, the characterization and differentiation of *TYMS*^−/−^-hiPSCs show that they provide a functional source for cell therapy that does not sustain unwanted proliferation without external dTMP supplementation.

## Results

### Disruption of *TYMS* makes proliferative cells dependent on thymidine supplementation

The human thymidylate synthase (PDB: 5X5D) is evolutionarily highly conserved. The functional enzymatic ligand pocket is encoded by exons 4 (amino acid R175), 5 (P193, C195, Q214, S216, D218, R225), and 6 (H256, Y258).^27^ To disrupt *TYMS* activity, we deleted the exon 5 with a dual single-guide RNA (sgRNA) Cas9 knockout approach targeting both intron 4 (I4) and I5 (Figure 1B). This removed the active site of the enzyme, generated an early stop codon in exon 6, and depleted the enzyme expression at both mRNA and protein levels (Figures 1C, 1D, S1A, and S1B) in all tested cell lines.

We hypothesized that *TYMS*^*−/−*^ cells could be cultured and manufactured normally with external dTMP supplementation, but they would cease to proliferate in its absence. Therefore, we followed the confluency of *TYMS*^−/−^ cancer cell lines and hiPSCs (HEL24.3)^28^ without or with dTMP supplementation (25 μM). The untreated (*TYMS*^+/+^) cell lines were used as controls. The results suggest that *TYMS*^*−/−*^ cells proliferate at a rate comparable to that of *TYMS*^+/+^ when supplemented with thymidine, while the proliferation capacity of non-supplemented *TYMS*^*−/−*^ cells was drastically impaired (Figures 1E and 1F). To determine the optimal concentration of dTMP for culturing *TYMS*^−/−^-hiPSCs, we ran a dose-dependent assay using a range of 0.3 μM–5 mM exogenous dTMP to explore their tolerance limits (Figures 1G and S1C). Contrarily to *TYMS*^+/+^ lines, *TYMS*^−/−^-hiPSCs could not sustain growth at physiological concentrations of thymidine (reference: 0.5–1.4 μM),^29^ and by day 5 had largely died, with only a few differentiated cells remaining (Figure S1D). On the other end of this spectrum, we observe a shift in the cytotoxic threshold at higher concentrations; while the growth rate of *TYMS*^+/+^ cells decreases at concentrations higher than 200 μM, knockout cells suffer comparable cytotoxic effects at concentrations higher than 1 mM (1,000 μM) (Figure 1F). For the hiPSCs we employed throughout this article (HEL24.3), we confirmed these results by quantifying the cell number of *TYMS*^−/−^ and *TYMS*^+/+^ at 24 (1 day) and 144 (6 days) after plating with or without 20 μM dTMP (Figure 1H). In yet another proof-of-concept experiment, we plated *TYMS*^−/−^ and *TYMS*^+/+^-hiPSCs in 48-well plates as single cells and followed their expansion with and without thymidine (Figures 1I and 1J). While all *TYMS*^+/+^ cells and supplemented *TYMS*^−/−^ reached 90% confluency between days 15 and 27 postplating, the few non-supplemented *TYMS*^−/−^ colonies differentiated early and eventually collapsed (data not shown). Cell-cycle analysis by flow cytometry of *TYMS*^−/−^-hiPSCs reveals that in the first 24 h of dTMP withdrawal, the cells accumulate in G0–G1 and S phase, and then, as they should continue to G2, accumulate in the subG0–G1 phase (Figures 1G and S1E). qRT-PCR of *CDKN1A* (P21) suggests the cells largely die during the first 24 h after supplementation withdrawal (Figure S1F).

In summary, our results show that *TYMS*^−/−^ cells grow normally and can be expanded extensively under dTMP supplementation. Thymidine withdrawal, however, stalls cells in S phase and inhibits progression to G2/M, likely due to replicative stress from nucleotide imbalance.

### *TYMS* knockout leads to few transcriptomic and metabolic changes

We explored the molecular effects of the genetic knockout through bulk RNA sequencing (RNA-seq) and untargeted metabolomic analysis of the supplemented *TYMS*^−/−^ and non-supplemented *TYMS*^+/+^-hiPSCs. The results of the RNA-seq further confirmed the downregulation of *TYMS* expression and identified some undescribed transcripts that might be playing a role in the gene regulation (e.g., LINC02864, ENSG00000286456) (Figures S2A and S2B). Enrichment analysis suggested changes in ion binding, mostly due to changes in metallothioneins of class MT1 (Figure S2C). These changes might relate to response to stimuli,^30^ likely linked to metabolic changes from thymidine supplementation. Interestingly, the results also showed a downregulation of CDKN1A (p21) in *TYMS*^−/−^ compared to *TYMS*^+/+^ (Figures S2B and S2C). The metabolomic analysis exposes a downregulation of thymidine subproducts (e.g., thymine), and an upregulation of metabolites that directly or indirectly participate in thymidine synthesis (Figures S2D–S2F and S3A). Taken together, these analyses show little difference between *TYMS*^−/−^ and *TYMS*^+/+^. Moreover, the differences seem to arise as a response to increased dTMP, supported by the upregulation of genes like MAT2A, FBXO9, and PPP1R17. These genes act on cell-cycle protein dynamics and methionine metabolism,^31^^,^^32^^,^^33^ which in turn may lead to the upregulation of AMP, anserine, hypoxanthine, *N*-methylglutamate, and *N*,*N*-dimethylarginine.

### *TYMS* knockout hiPSCs maintain pluripotency and genomic integrity

**Figure 2:**
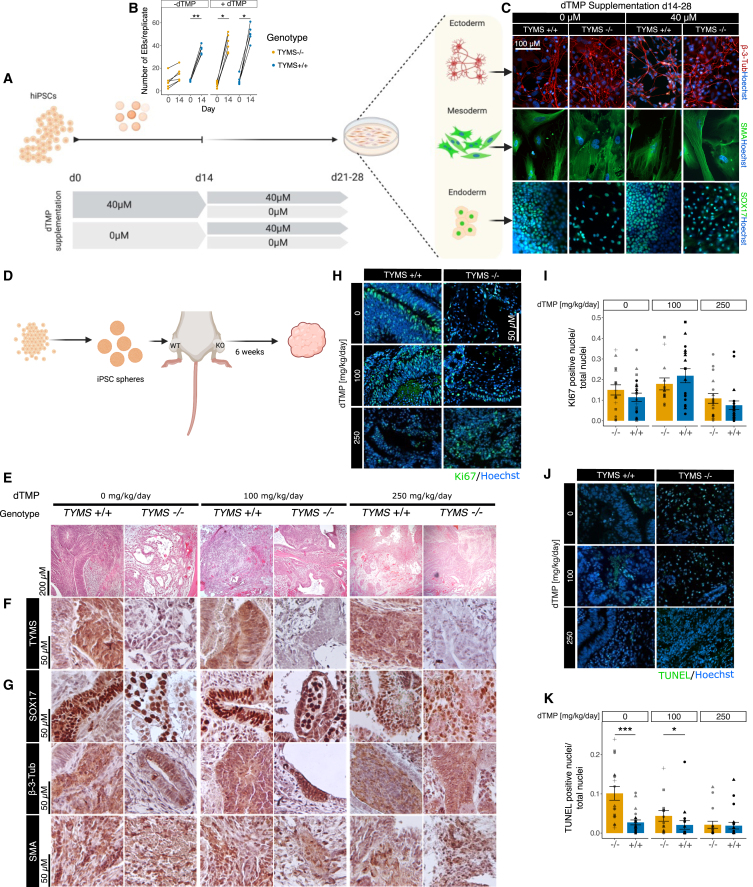
Thymidine deficiency drastically reduces proliferation and teratoma formation in mice transplanted with *TYMS*^−/−^-hiPSCs (A) Graphical representation of trilineage differentiation protocol. (B) Number of resulting 300-μm aggregates from wild-type and knockout hiPSC with and without supplementation during the first stage of differentiation (days 0–14). (C) Immunocytochemistry analysis of markers for ectoderm (β-3-tubulin), mesoderm (smooth muscle actin, SMA), and endoderm (SOX17) in cells derived from wild-type and knockout hiPSCs under dTMP supplementation during the first stage of differentiation. (D) Graphical representation of teratoma formation experiment. (E) H&E staining showing dense and cystic structures within the tumors. (F) Immunohistochemistry against *TYMS*. (G) Immunohistochemistry of characteristic markers of endoderm (SOX17), ectoderm (β-3-tubulin), and mesoderm (SMA). (H) Immunohistochemistry against Ki67 (proliferation marker, green) and total nuclei (Hoechst, blue). (I) Quantification of Ki67 index from immunohistochemistry, shown as positive nuclei over total nuclei. (J) TUNEL assay (apoptotic cells, green) and total nuclei (Hoechst, blue). (K) Quantification of TUNEL^+^ nuclei over total nuclei. Statistical significance in (B), (I), and (K) based on Wilcoxon test; ns *p* > 0.05; ∗*p* < 0.05; ∗∗*p* < 0.01; ∗∗∗*p* < 0.001; ∗∗∗∗*p* < 0.0001. See also Figure S3.

The *TYMS*^−/−^-hiPSC lines performed equally well on standard pluripotency analysis as their non-edited counterparts. They showed positive staining for pluripotency markers NANOG, OCT4, SOX2, and SSEA4 and comparable mRNA expression of *SOX2*, *NANOG*, and *OCT4* to *TYMS*^+/+^-hiPSC (Figures S1G and S1H). Furthermore, their chromosomal integrity remained unaltered by the editing process in prolonged culture, tested in several time points for up to 25 passages (Figure S1I). The differentiation into the three germ layers with or without dTMP supplementation revealed that the cells successfully differentiate into ectoderm, mesoderm, and endoderm when supplemented with dTMP at the early stages of differentiation (Figures 2A–2C). Lack of supplementation from the *TYMS*^−/−^ cells during days 0–14 produced significantly less embryoid bodies at day 14 (Figure 2B). By day 28, however, all attached cells from supplemented and non-supplemented cells expressed characteristic markers of the three germ layers (Figures 2C and S3B).

### Thymidine deficiency drastically reduces proliferation and teratoma formation in mice transplanted with TYMS^−/−^-hiPSCs

Next, we aimed to translate the *in vitro* trilineage differentiation to an *in vivo* setting. We implanted undifferentiated *TYMS*^*+/+*^- and *TYMS*^*−/−*^-hiPSC aggregates subcutaneously in mice, without or with dTMP supplementation (100 and 250 mg/kg/day) in the drinking water (Figure 2D). After 6 weeks, we retrieved the teratomas from the euthanized mice and analyzed their composition and proliferation (Figures 2E–2K).

*TYMS*^+/+^-hiPSC produced teratoma-like growth in all mice regardless of the thymidine supplementation, while *TYMS*^*−/−*^-hiPSCs produced visible teratomas only under dTMP supplementation. Only one mouse presented a small *TYMS*^*−/−*^-derived teratoma without supplementation (Figure 2E), composed mainly of primitive glial, epithelial, and mesenchymal tissue. As expected, immunohistochemical analysis of *TYMS*^−/−^-hiPSC-derived tumors was negative for TYMS, while *TYMS*^+/+^-hiPSCs present a rather homogeneous expression of the protein (Figure 2F). Additionally, analogous to the *in vitro* experiment, we could identify regions expressing markers for the three germ layers (Figure 2G) in all analyzed tumors. Importantly, although we did not find differences in the Ki67 index (Ki67^+^ nuclei over total nuclei) (Figures 2H and 2I), likely due to the reduced cell density in non-supplemented *TYMS*^−/−^ cells (Figure S3C), we observed a significantly higher ratio of apoptotic cells from the TUNEL assay (as TUNEL^+^ nuclei over total nuclei) (Figures 2J and 2K).

In summary, we found that *TYMS*^−/−^ cells produce proliferative teratomas *in vivo*, similar to those obtained with *TYMS*^+/+^ cells only under dTMP supplementation. Without it, however, the teratomas present lower cellular density and an increased percentage of apoptotic cells.

### TYMS^−/−^-hiPSC cultures efficiently terminally differentiate *in vitro*

**Figure 3:**
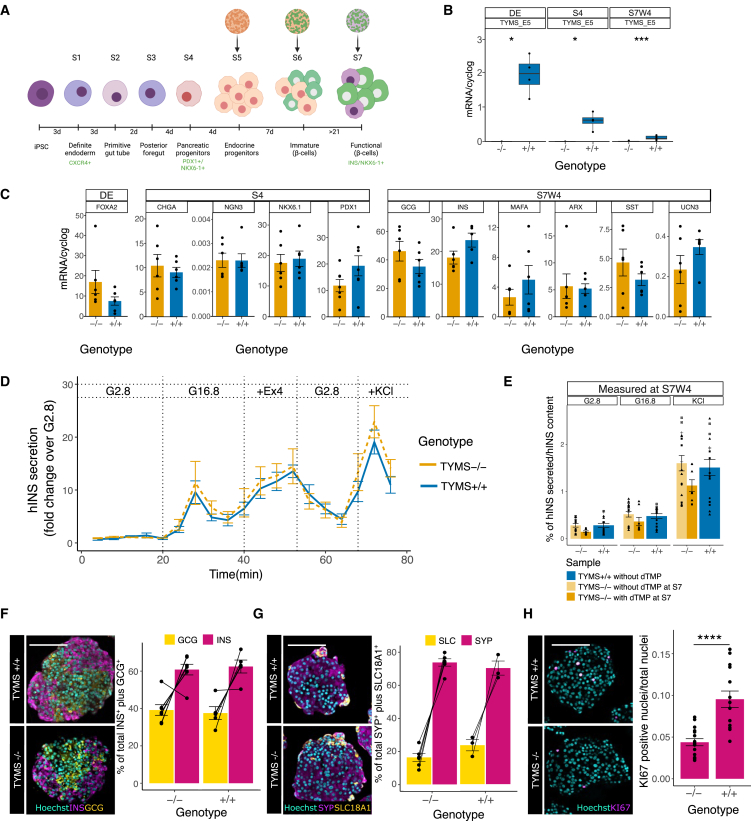
*TYMS*^−/−^-hiPSC cultures efficiently terminally differentiate *in vitro* (A) Graphical representation of *in vitro* β cell differentiation from hiPSCs. Most tests were carried out S7W4, after 4 weeks from dTMP withdrawal. (B) mRNA expression analysis of *TYMS* (primers target exon 5) at different stages of differentiation. Results shown as ratio of *TYMS* mRNA over GAPDH mRNA. (C) mRNA expression analysis of maturation markers at different stages. Results shown as ratio of marker mRNA over GAPDH mRNA. (D) Dynamic insulin secretion in response to different stimuli at S7W4. (E) *In vitro* insulin secretion in response to low (2.8) and high (16.8) glucose concentration and glucose plus KCl (2.8 KCl) at S7W4 (E). (F) Immunohistochemistry and quantification of INS (magenta) and GCG (yellow) in hiPSC-derived islets at S7W4. (G) Immunohistochemistry and quantification of SYP (magenta) and SLC18A1 (yellow) in hiPSC-derived islets at S7W4. (H) Immunohistochemistry of Ki67 (magenta) in hiPSC-derived islets at S7W4 and quantification of proliferation index, as number of Ki67^+^ nuclei over the total nuclei. Statistical significance based on Wilcoxon test; ns *p* > 0.05; ∗*p* < 0.05; ∗∗*p* < 0.01; ∗∗∗∗*p* < 0.0001. Scale bar, 100 μM. See also Figure S4.

Once we ensured that the genetic disruption did not affect the pluripotency of our stem cells, we explored their directed differentiation into a potentially therapeutic cell type. In this context, diabetes represents a promising candidate for cell replacement therapy, in which stem cell-derived insulin-producing β cells act as the therapeutic source of insulin. We evaluated the safety, quality, and function of *TYMS*^−/−^-hiPSC-derived pancreatic islets using a well-defined, multi-step protocol for β cell differentiation (Figure 3A).^5^^,^^6^

We differentiated *TYMS*^−/−^ -hiPSCs (HEL24.3) *in vitro*, supplemented with thymidine (40 μM) through the whole differentiation experiment. Meanwhile, as expected, non-supplemented *TYMS*^−/−^ cells did not survive thymidine withdrawal at early stages of differentiation, making further characterization of this condition impossible. Non-supplemented *TYMS*^+/+^-hiPSCs were used as controls. We did not observe differences between *TYMS*^+/+^-hiPSCs differentiated with or without dTMP supplementation, suggesting that the addition of dTMP at our working concentrations (up to 40 μM) does not affect *TYMS*^+/+^ cells (data not shown). Hereafter, we did not supplement *TYMS*^+/+^-hiPSCs during the differentiation.

We evaluated the progression of the differentiation by flow cytometry, qRT-PCR, and immunocytochemical analysis of cell identity markers at several developmental stages. With qRT-PCR analysis, we found that *TYMS*^−/−^-derived islets presented lower (close to zero) *TYMS* mRNA levels than their *TYMS*^+/+^ counterparts across all stages (Figure 3B). Nevertheless, both the *TYMS*^−/−^- and *TYMS*^+/+^-derived islets expressed similar levels of markers at stages definitive endoderm (DE) and pancreatic endocrine progenitor (S4), and markers of mature β cells at week 4 of stage 7 (S7W4) (Figure 3C). Accordingly, *TYMS*^+/+^- and supplemented *TYMS*^−/−^-hiPSCs showed comparable *in vitro* expression levels of markers at the DE stage (CXCR4), S4 (PDX1, NKX6.1), and endocrine maturation stage (S7) by flow cytometry and immunocytochemistry (Figure S4A–S4D).

### dTMP withdrawal from differentiated TYMS^−/−^-β cell does not affect their function

**Figure 4:**
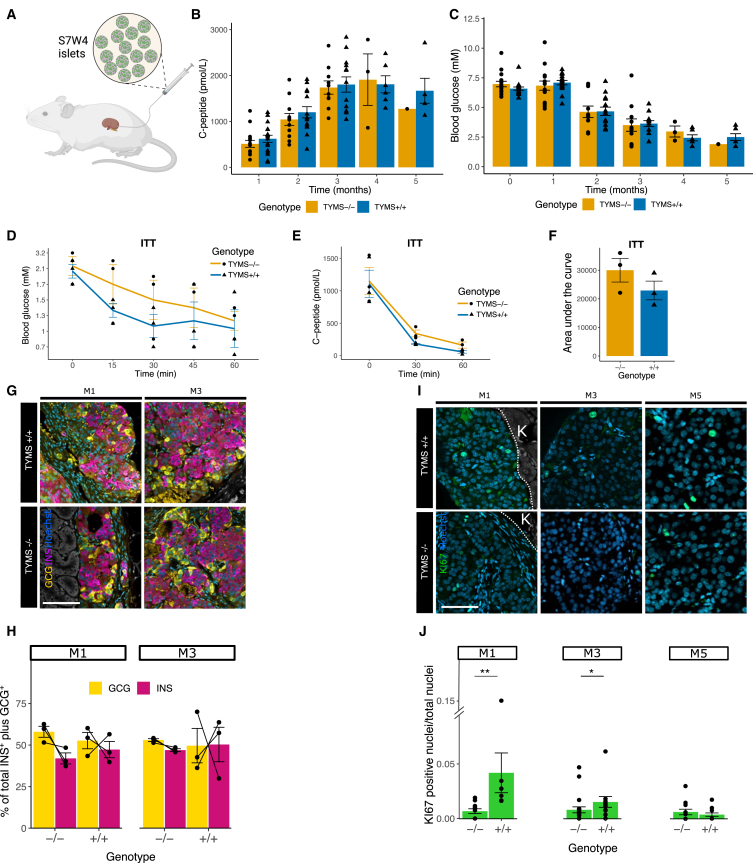
*TYMS*^−/−^-hiPSC-derived β cells without dTMP supplementation efficiently regulate blood glucose *in vivo* (A) Diagram of S7W4 islet implantation setup. (B and C) Follow-up of human C-peptide secretion (in serum) and blood glucose in mice up to 5 months from implantation (month 0). (D) Blood glucose of mice under ITT at every 15 min for 1 h, at month 3. (E) Human C-peptide secretion (in serum) during ITT before and after 30 min and 1 h from insulin administration. (F) Quantification of area under the curves of human C-peptide secretion. (G) Immunohistochemistry of engrafted kidney 1 and 3 months postimplantation. Insulin (INS) (magenta), glucagon (GCG) (yellow). (H) Quantification of INS and GCG area from the islets in (G). (I) Immunohistochemistry against Ki67 in grafts after 1, 3, and 5 months. (J) Proliferation index, based on the quantification of Ki67^+^ nuclei over the total number of nuclei from (I). Statistical significance based on Wilcoxon test; ns *p* > 0.05; ∗*p* < 0.05; ∗∗*p* < 0.01. K, kidney. Scale bar, 50 μM. See also Figure S4.

At S7, we withdrew dTMP supplementation from the islets cultured for *in vivo* implantation. By the last stage before implantation (S7W4), *TYMS*^−/−^ and *TYMS*^+/+^ hiPSC-derived islets showed similar *in vitro* levels of glucose-, exendin 4-, and KCl-induced functional insulin secretion at S7W4 (Figure 3D and 4E), and even after 3 months in culture (S7W16; Figure S4E). The *TYMS*^*−/−*^-hiPSC islets showed normal morphology and standard percentages of insulin, glucagon, synaptophysin (SYP), and SLC18A1-expressing cells (Figure 3F and 4G), while displaying a significantly decreased proliferative index by Ki67 analysis (Figure 3H). Thus, these results overall indicate that *TYMS*^−/−^-hiPSC lines efficiently differentiate to functional stem cell-derived (SC)-islets, comparable to their control counterparts, while displaying reduced proliferation upon dTMP withdrawal.

### *TYMS*^−/−^-hiPSC-derived β cells without dTMP supplementation efficiently regulate blood glucose *in vivo*

We next evaluated the function of mature *TYMS*^−/−^ β cells (S7) *in vivo* by engrafting them under the kidney capsule of immunosuppressed mice without dTMP supplementation (Figure 4A). We implanted mice with *TYMS*^+/+^- or *TYMS*^−/−^-hiPSC-derived islets, and monitored their body weight, glucose levels, and the grafts’ production of human C-peptide for 5 months.

Both *TYMS*^−/−^ and *TYMS*^+/+^ control islets produced comparable levels of human C-peptide and lowered the mouse blood glucose (Figures 4B and 4C). All mice maintained their normal body weights (Figure S4F) and did not show any other signs of health concerns.

Three months postimplantation, we performed an insulin tolerance test (ITT) by treating the mice with insulin to test the ability of the grafts to shut down their bona fide insulin secretion in response to low glucose levels. Upon the decline of circulating glucose levels in response to the exogenously injected insulin (Figure 4D), our *TYMS*^−/−^ grafts stopped their insulin secretion at rates comparable to those of their *TYMS*^+/+^ counterparts, suggesting an appropriate regulation of this process (Figures 4E and 4F).

Immunohistochemistry on the implanted grafts showed a comparable percentage of insulin- and glucagon-producing cells in *TYMS*^+/+^ and *TYMS*^−/−^ grafts at 1, 3, and 5 months postimplantation (Figures 4G and 4H).

Additionally, we ran an immunohistochemical analysis against Ki67 to quantify the proliferation of the cells in the grafts *in vivo* through time (1, 3, and 5 months). Interestingly, despite finding no significant differences in functionality, we did observe a reduction in active proliferation of *TYMS*^−/−^ cells (by Ki67 index) during the first 3 months after implantation (Figures 4I, 4J, and S4G).

To summarize, we demonstrate here that *TYMS*^−/−^-hiPSCs differentiate into pancreatic β cells and function normally *in vitro* and *in vivo*, as seen by prolonged regulated human insulin secretion.

## Discussion

We have developed a method to generate controllable cells without inserting any external genetic elements. By disrupting the rate-limiting DNA-specific nucleotide synthesis reaction, we have obtained cells auxotrophic to thymidine. This novel safety mechanism allows *TYMS*^−/−^-hiPSCs to expand for undefined periods showing no signs of exhaustion, but only when externally supplemented with thymidine. In its absence, contrarily, the cells fail to proliferate. Moreover, this method does not affect the function of the therapeutic differentiated cells *in vitro* or *in vivo*.

The *TYMS*^−/−^-hiPSCs present only a few transcriptomic and metabolomic differences with their wild-type counterpart, largely related to the metabolism of the supplemented dTMP. They retain their pluripotency and differentiate successfully into different cell types. Non-supplemented *TYMS*^−/−^-hiPSCs generate small or no teratomas *in vivo* with a significantly increased percentage of apoptotic cells (TUNEL), pointing to the gradual loss of proliferative cells under physiological concentrations of thymidine. Previous reports by Diehl et al.^34^ suggest that thymidine withdrawal from *TYMS*^−/−^ hiPSCs leads to replicative stress and cell-cycle arrest from nucleotide starvation. Indeed, dTMP withdrawal quickly affects the cell cycle of hiPSCs, with a concurrent increase in p21 expression, suggesting the activation of apoptosis processes or cell-cycle arrest.^35^^,^^36^ Supported by the results of our cell-cycle analysis, we hypothesize that cells that have not exited the cell cycle likely die soon after this replicative stress is sensed, at the S-G2 checkpoint.^34^^,^^37^ However, we observed an extended survival time of non-supplemented *TYMS*^−/−^ cells when they form three-dimensional aggregates both in *in vitro* and *in vivo* differentiation experiments (embryoid bodies and teratoma formation, respectively). As *TYMS* activity is required for the synthesis of DNA but not that of RNA, this may hint at the recycling of thymidine from apoptotic cells until cell-cycle exit.^38^ In combination with the Ki67 analysis, the TUNEL assay on our teratomas may indeed suggest an active degradation of cells, which in turn could serve as a thymidine source for the Ki67^+^ cells in the absence of external supplementation.

Once out of the cell cycle, the cells may survive as differentiated progeny for extended periods. Accordingly, we see no functional alterations in the terminally differentiated cells without dTMP supplementation *in vitro* or *in vivo*. The hiPSC-derived pancreatic islets survived implantation and proved to successfully secrete human insulin and lower blood glucose up to the measured 5 months after implantation, without affecting the overall well-being of the mice.

The advances in basic science and technological developments simultaneously aid and challenge the development of clinical applications. On the one hand, improved gene and cell manipulation protocols have increased the efficiency and widened the applications of somatic cell and gene therapies, tissue-engineered medicines, and CRISPR-based products. On the other hand, the safety concerns of therapeutic cells with altered DNA challenge further clinical applications. Hence, strategies to make cellular products safer become a need. Ideally, a safety mechanism for cell therapies selectively targets cells that retain or have acquired the ability for dangerous proliferation, without affecting the functional therapeutic cells. Although attempts to control the activity and proliferation of the therapeutic cells have proven relatively efficient, they present some inherent disadvantages. As most safety switches alter DNA by including exogenous genetic material, they often present as complex, immunogenic, or susceptible to mutations or silencing, ultimately affecting the partial/remissive function of the system.^25^

In this regard, we believe our system brings added value to the efficiency and versatility of safety mechanisms. By a simple genetic manipulation of endogenous DNA, we generated cells auxotrophic toward thymidine. This approach not only evades the insertion of external genetic sequences but it also allows the control of cellular proliferation by the addition of a simple compound (thymidine). Thus, it facilitates the mass expansion and generation of therapeutic cells, which lose their proliferative capacity, but not their function, under *in vivo* concentrations of thymidine. Moreover, *in vivo* thymidine administration may control the function and efficiency of some therapeutic cells, such as immunotherapies, expanding the applications of this strategy.

The genetic disruption applied in this research brings about an additional advantage: the use of the *TYMS* locus as a genomic safe harbor. This opens the doors to future additions and combinatory developments with other selection and safety systems. We see this versatile feature as a solution to current limitations and an aid to expand the applications of *TYMS* disruption to other aims and other therapeutic cell types. We expect the replication and utilization of these results to contribute to the optimization of hiPSC-derived and other cellular products and to the future development of safer efficient cell-based therapies.

## Materials and methods

### Culture of A431, A549, HSC3

We cultured epidermoid carcinoma cell line A431 (RRID: CVCL_0037), adenocarcinoma human alveolar basal epithelial cells A549 (RRID: CVCL_0023), and human oral squamous carcinoma cell line HSC3 (RRID: CVCL_1288) cells in DMEM containing 10% fetal bovine serum (FBS), 100 μg/mL penicillin-streptomycin, and 2 mM GlutaMAX (all Thermo Fisher Scientific). After TYMS knockout, cell media were additionally supplemented with 5–20 μM dTMP (Thermo Fisher Scientific). We kept the cell lines at 37°C and 5% CO_2_, with media change every other day until splitting with TrypLE select (Gibco). All cell lines tested negative for mycoplasma.

### IPSCs culture and characterization

The hiPSC line HEL24.3 (RRID: CVCL_9T96) was cultured on Matrigel (Corning)-coated plates in E8 medium (Thermo Fisher Scientific) and split using 0.5 mM EDTA. We kept the cell lines at 37°C and 5% CO_2_, with media change every other day. All cell lines tested negative for mycoplasma. After TYMS knockout, cell media were additionally supplemented with 5–20 μM dTMP. The karyotyping was carried out by Ambar Anàlisis Mèdiques (Barcelona, Spain) by G-banding. We treated edited (passages 20 and 45) and non-edited (passage 20) hiPSCs with Colcemid for 4 h, 37°C, 5% CO_2_ and prepared the cells as recommended by the service provider.

### Genome editing

To knock out exon 5, we designed gRNA targeting I4 and I5 using online tools (https://benchling.com, CRISPOR^39^; Table S1). We generated the sgRNA by incubating our gRNA (customized Alt-R CRISPR-Cas9 gRNA, Integrated DNA Technologies [iDT]) for 5 min at 95°C with Alt-R CRISPR-Cas9 tracrRNA, ATTO 550 (iDT).

For each stem cell electroporation experiment, we dissociated 2 × 10^6^ cells into single cells with StemPro Accutase (Thermo Fisher Scientific). We complexed the sgAlt-R S.p. HiFi Cas9 Nuclease V3 and both sgRNAs to form the functional ribonucleoprotein (RNP) and delivered this RNP into the cells, along the Alt-R Cas9 electroporation enhancer (all from iDT), by electroporation with Neon transfection systems (1,100 V, 20 ms, 2 pulses).

Cells were plated onto Matrigel-coated plates containing E8 with 5 μM ROCK inhibitor (Y-27632, Selleckchem) and 20–40 μM dTMP and incubated at 37°C, 5% CO_2_.

For the electroporation experiment with cell lines HSC-3, A549, and A431, cells were dissociated into single cells with TrypLE. sgRNAs were prepared as previously described and delivered to the cells using Neon transfection systems. For HSC3 and A549, the electroporation settings were 1250 V, 10 ms, 1 pulse, while for A431 we used 1450 V, 20 ms, 2 pulses. After transfection, the cells were plated onto plates containing culture media (10% FBS in DMEM) supplemented with 20–40 μM dTMP and incubated at 37°C, 5% CO_2_.

At 24–48 h after electroporation, we single-cell sorted ATTO550^+^ cells for monoclonal expansion in 96-well plates containing their corresponding dTMP-supplemented culture media (hiPSCs additionally supplemented with 10% Clone R [STEMCELL Technologies]). The media were refreshed every 72 h until splitting. We individually screened monoclonal colonies by PCR. All PCR products of the selected edited clones were validated by Sanger sequencing, along with the integrity of the top seven off-target sequences (Table S2).

### Embryoid body (EB) differentiation

To test the pluripotency for an embryonal lineage differentiation, we grew the cells to 90%–100% confluency and performed an EB) differentiation assay as previously described.^2^ The final plated EBs were fixed with 4% paraformaldehyde (PFA)-PBS and analyzed by immunocytochemistry for characteristic markers of the three germ layers (see list below).

### Teratoma assay

Knockout and wild-type HEL24.3 cells were cultured in ultra-low attachment plates, with E8 supplemented with 5 μM ROCK inhibitor for 24–48 h to form aggregates. The medium for knockout cells was additionally supplemented with 40 μM dTMP until 2 h before implantation. Groups of approximately 500 spheres were collected in syringe cannulas for subcutaneous implantation in the legs of the mice. The drinking water was supplemented with 0, 100, or 250 mg/kg/day dTMP (Table S3). At 6–8 weeks postimplantation, the teratomas were extracted and fixed for immunocytochemistry and H&E staining as described below.

### dTMP auxotrophy

Wild-type and knock-out lines from each tested cell line were split onto Matrigel-coated 6-well plates at a reason of a 25,000 cells per well. Each well contained the corresponding medium for the cell line (see Culture of A431, A549, HSC3 and IPSCs culture and characterization) supplemented with 0–5 mM dTMP. The growth of the cells was followed using IncuCyte imaging systems (Sartorius), taking 49 pictures per well per time point. Cells were washed and media were refreshed daily to clear away dead cells debris and keep the thymidine concentration as constant as possible. For statistical significance, 3 biological replicates were plated per condition and 20 random images per well per time point were considered for quantification. The results are shown as average confluency per image ±SE.

### β cell differentiation

The *in vitro* β cell differentiation was carried out as previously described.^5^^,^^6^ Briefly, *TYMS* knockout and control HEL24.3 hiPSCs were seeded onto Matrigel-coated 10-cm plates, as previously stated. The differentiation protocol was started 24 h postseeding, when the medium was changed to D0 medium. Both cell lines were supplemented with 0 or 40 μM dTMP until maturation. At S7, dTMP was withdrawn or kept until analysis (weeks 4–16 from the first day of S7). The media were refreshed every 2–3 days until analysis. Three independent rounds of differentiation were conducted, including 3–5 biological replicates per genotype each.

### Flow cytometry

S4 cells and S7 SC-islets were dissociated with TrypLE for 10 min at 37°C and resuspended in 5% FBS-containing PBS. Fixation and permeabilization were done using Cytofix/Cytoperm (BD Biosciences, catalog no. 554714) for 20 min at room temperature (RT). Then, samples were incubated overnight with primary antibodies at 4°C, followed by secondary antibodies for 30 min at RT in Perm/Wash buffer (BD Biosciences, catalog no. 554714) supplemented with 5% FBS. The cells were run on the FACSCalibur cytometer (BD Biosciences); data were collected with CellQuest Pro version 4.0.2 (BD Biosciences) and analyzed with FlowJo version 10.8 software (BD Life Sciences). The antibodies are listed in Table S4.

For cell-cycle analysis, wild-type and knockout hiPSCs were plated overnight on 6-well plates containing E8 supplemented with ROCK inhibitor and 20 μM dTMP. Then, the media were refreshed, and for some plates, the dTMP supplementation was stopped. Cells from individual wells were collected at 0, 2, 4, 10, 16, and 24 h after dTMP withdrawal and fixed using 4% PFA in PBS. The same time points were collected for the supplemented plates. After fixation, the samples were washed and incubated 30 min with 10 μg/mL DAPI diluted in 1% Triton X-100 (Sigma-Aldrich) in PBS. Then, they were immediately analyzed using the NovoCyte Quanteon 4025.

### Insulin secretion analysis *in vitro*

For the static analysis of insulin secretion, a total of 30–50 SC-islets were picked and incubated for 90 min in a 12-well plate containing 2.8-mM glucose (G2.8) in Krebs-Ringer buffer (KRB) for equilibration. This was followed by sequential 30-min incubations of G2.8, 16.8 mM glucose (G16.8) and G2.8 + 30 mM KCl in KRB. After each incubation, 200-μL samples were taken for further insulin secretion analysis. Once all samples were taken, the islets were collected for DNA quantification. Dynamic insulin secretion test was carried out using a perifusion apparatus (Brandel Suprafusion SF-06) at a 0.25-mL min^−1^ flow rate, sampling every 4  min. A total of 50 SC-islets were handpicked and perfused with KRB; the sample collection started after 90 min of equilibration in G2.8. The insulin content of secretion fractions and SC-islet lysates was analyzed with ELISA (Mercodia).

### Islet *in vivo* characterization

Animal care and experiments were approved by the National Animal Experiment Board in Finland (ESAVI/9734/2021). NOD-SCID-Gamma (NSG, Jackson Laboratories, catalog no. 0055577) mice were housed in the Biomedicum Helsinki conventional facility in 12-h light/dark cycle and fed standard chow. SC-islet implantations were done following a previously established described protocol.^4^ A total of 32 mice between 6 and 18 months old were used in 3 individual implantation experiments, each including islets from 3 differentiation experiment replicates. Non-fasted blood samples were collected monthly from the saphenous vein for glucose measurement and C-peptide secretion analysis. For further characterization of the SC-islet graft, the engrafted kidney was removed after 1 or 3 months and processed as described below.

For the ITT at 3 months postimplantation, 3 mice implanted with *TYMS*^−/−^ and 3 with *TYMS*^+/+^ islets were weighted and injected with insulin (0.75 IU/kg). Blood glucose was measured every 15 min for 1 h and serum samples for human C-peptide secretion analysis were measured at 30 and 60 min.

### H&E staining

Teratoma-like growths were fixed using 4% PFA-PBS overnight and then embedded in paraffin and sectioned. Dried sections were deparaffinized and stained using a standard protocol. Briefly, they were deparaffinized using xylene (3 × 10 min) and gradually hydrated using decreasing concentrations of ethanol (99% EtOH 3 × 4 min, 96% EtOH 1 × 4 min, 70% EtOH 1 × 2 min). Then, they were incubated in hematoxylin for 2.5–3 min, washed in indirect flowing tap water for 5–10 min, followed by a 1-min incubation in ultrapure water. The samples were incubated in eosin for 2 min and dehydrated using increasing concentrations of ethanol (96% EtOH 3 × 10 s, 99% EtOH 2 × 2 min) and xylene (2 × 4 min). Slides were mounted with coverslips using mounting media and imaged the next day.

### Immunocytochemistry, immunohistochemistry, and image analysis

Samples from hiPSCs were fixed using 4% PFA-PBS for 20 min and permeabilized using 1% Triton X-100 in PBS. Samples from S4 and S7 SC-islets were fixed with 4% PFA in PBS for 2 h, and explanted SC-islet grafts and teratoma-like growths were fixed overnight. After fixation, these samples were embedded in paraffin and sectioned.

For immunohistochemistry and immunocytochemistry, 5-μm sections were deparaffinized and subjected to heat-induced antigen retrieval in 0.1 mmol l–1 citrate buffer. The cells and tissue slides were blocked with UV-block (Thermo Scientific, catalog no. TA-125-PBQ) and incubated with primary antibodies in 0.1% Tween 20 overnight at 4°C with the given dilutions (Table S4). After washing, secondary antibodies diluted in a similar manner were incubated at RT for 1 h. For immunocytochemistry, secondary antibody incubation was done in the presence of Hoechst 33342 for nuclear staining. For immunohistochemistry, after the secondary antibody, the slides were incubated with a 3,3′-diaminobenzidine solution for 2–10 min until a brown color developed. Then, they were counterstained with hematoxylin for 2.5–3 min and dehydrated following a standard protocol (see “H&E staining” above).

The cells and immunocytochemistry slides were imaged using Apotome II from a Zeiss AxioImager at the Biomedicum Imaging Unit, with the same exposure and export setting used on all slides of each targeted marker. Immunohistochemistry slides were imaged using an Olympus BX51 microscope.

Images were processed in Zen2 Blue Edition version 2 (Zeiss) and analyzed using Fiji^40^ with pipelines adapted from a previous report.^41^ The same pipeline settings were used on all images of each targeted marker.

### Protein expression analysis

mRNA expression levels were analyzed by qRT-PCR on three biological replicates (Tables S5–S7). TYMS protein expression was assessed by standard western blot. Briefly, protein was extracted by using radioimmunoprecipitation assay lysis buffer (20–188, Millipore) supplemented with cOmplete Mini protease and phosphatase inhibitors tablets (Roche). Samples were sonicated (30 s, 50% duty cycle pulse) and centrifuged for soluble protein extraction. Protein quantification was done using a Pierce BCA protein assay kit (Thermo Fisher), and 20- to 30-μg total protein were run in Mini-PROTEAN TGX Precast gels (4561033, Bio-Rad). Transfer to a nitrocellulose membrane was done using 25 V for 6 min in the iBlot2 Gel Transfer device (IB21001, ThermoFisher). Then, the membrane was probed with a rabbit polyclonal primary antibody against *TYMS* (1:2,500, Proteintech; Table S4). For detection, we used an anti-rabbit horseradish peroxidase (HRP)-coupled secondary antibody (1:5,000, Cell Signaling Technology) and Clarity Western ECL Substrate (1705061, Bio-Rad). As a loading control, we probed the membrane with a β-actin-HRP-coupled antibody (sc-47778, Santa Cruz).

### Bulk RNA-seq

The 3′primed RNA-seq service was provided by the Biomedicum Functional Genomics Unit (FuGu) at the Helsinki Institute of Life Science and Biocenter Finland at the University of Helsinki. Wild-type and supplemented *TYMS* knockout cells were seeded in Matrigel-coated 6-well plates and expanded to 70%–80% confluency. Cells of 6 replicates each were washed twice with PBS, lysed, and collected using lipopolysaccharide binding protein (LBP, 740906.125, Macherey-Nagel), and purified using a NucleoSpin RNA isolation kit (Macherey-Nagel). Quality control, Illumina sequencing, and processing of the raw data were carried out by FuGu. Normalization and differential expression was done in R using the DESeq2 package^42^; enrichment was carried out using g:Profiler.^43^

### Metabolomics analysis

Wild-type and supplemented *TYMS* knockout cells were seeded in Matrigel-coated 6-well plates and expanded to 70%–80% confluency. Cells from 5 replicates per genotype were collected and analyzed on Thermo Q Exactive Focus Quadrupole Orbitrap mass spectrometer coupled with a Thermo Dionex UltiMate 3000 high-performance liquid chromatography (HPLC) system (Thermo Fisher Scientific). The HPLC was equipped with a hydrophilic ZIC-pHILIC column (150 × 2.1 mm, 5 μm) with a ZIC-pHILIC guard column (20 × 2.1 mm, 5 μm, Merck Sequant). A total of 5 μL of the samples were injected into the LC-mass spectrometry after quality controls in randomized order, having every tenth sample as blank. A linear solvent gradient was applied for separation, in decreasing organic solvent (80%–35%, 16 min) at 0.15 mL/min flow rate and 45°C column oven temperature. The mobile phases included aqueous 200 mmol/L ammonium bicarbonate solution (pH 9.3, adjusted with 25% ammonium hydroxide), 100% acetonitrile, and 100% water. The ammonium bicarbonate solution was kept at 10% throughout the run, resulting in a steady concentration of 20 mmol/L. Metabolites were analyzed using a mass spectrometer equipped with a heated electrospray ionization source using polarity switching and the following settings—resolution of 70,000 at *m/z* of 200; spray voltages: 3,400 V for positive mode and 3,000 V for negative mode; sheath gas: 28 a.u., auxiliary gas: 8 a.u.; temperature of the vaporizer: 280°C; and temperature of the ion transfer tube: 300°C. The instrument control was conducted with Xcalibur 4.1.31.9 software (Thermo Scientific). The peaks for metabolites were confirmed with commercial standards (Sigma-Aldrich). The data quality was monitored using a parallel in-house quality control cell line extracted similar to other samples. The final peak integration was undertaken with the TraceFinder 4.1 SP2 software (Thermo Scientific), and the peak area data were exported as a Microsoft Excel file for further analysis. The peak area of each metabolite was normalized to the sum of the absolute peak areas of all metabolites in the same sample. These data were then analyzed in R using the limma package,^44^ and enrichment analysis was run in MetaboAnalyst 6.0.^45^

### Statistical analyses

Statistical analyses for all figures were carried out in R. A non-parametric Wilcoxon test was performed to determine the *p* values and statistical significance for the comparisons between genotypes. Blinding of all samples (for immunocytochemistry, immunohistochemistry, serum samples, blood glucose measurements, etc.) was done by coding the sample names based on numbers (e.g., of paraffin block or animal). No results were excluded from analysis.

### Data and code availability

All main data are available in the main text, figures, or the supplemental information. Requests for reagents and cell lines used in this study should be directed to the corresponding authors.

## Supplemental information

Document S1. Figures S1–S4 and Tables S1–S7

Document S2. Article plus supplemental information
