# Supplementary material for: Thymidylate synthase disruption to limit cell proliferation in cell therapies
Source: Mol Ther. 2024 Jun 12;32(8):2535–48. doi: 10.1016/j.ymthe.2024.06.014 (PMC11405178; doi:10.1016/j.ymthe.2024.06.014)
Supplement: Document S1. Figures S1–S4 and Tables S1–S7 [file mmc1.pdf]

YMTHE, Volume *32*

Supplemental Information

Thymidylate synthase disruption to limit cell proliferation in cell therapies

Rocio Sartori-Maldonado, Hossam Montaser, Inkeri Soppa, Solja Eurola, Juhana Juutila, Melanie Balaz, Henri Puttonen, Timo Otonkoski, Jonna Saarimäki-Vire, and Kirmo Wartiovaara

Supplemental Materials

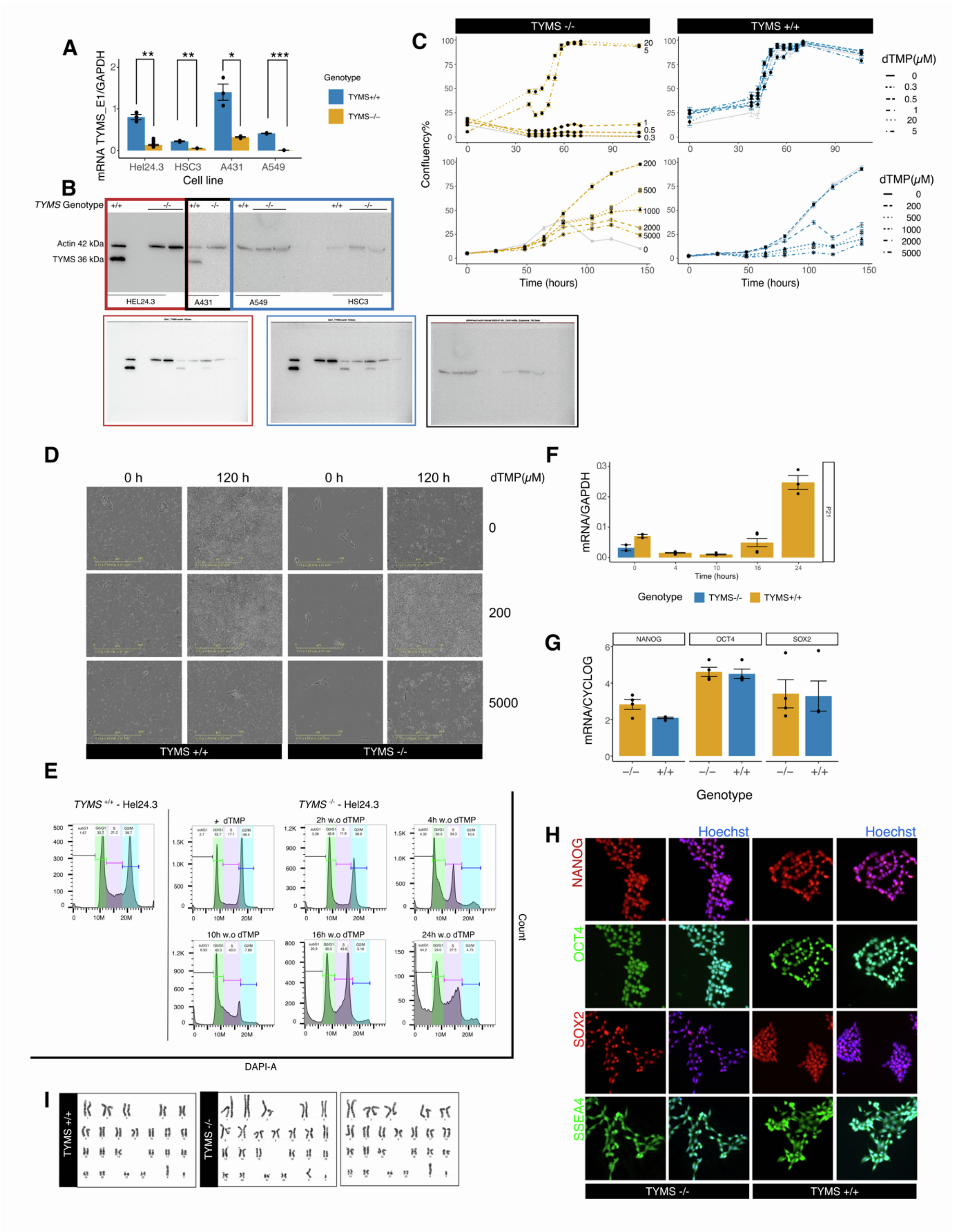

Figure S1. A. TYMS mRNA expression in wild-type versus knock-out cell lines (HEL24.3, A431, A549 and HSC3) using primers targeting exon 1 (n=3/cell line). Results shown as ratio of TYMS mRNA over GAPDH mRNA. B. Full western blot membranes of TYMS in wild-type and edited cell lines. C. Full growth curves of wild-type and knock-out hiPSC under different concentrations of dTMP (0 to 5000 µM). Results shown as average confluency per image ‡ SD. D. Representative pictures of wild-type and knock-out hiPSC supplemented with 0, 200, and 5000 µM dTMP at day 0 and 5. E. Cell cycle analysis of knock-out hiPSC during the first 24 h after dTMP withdrawal. F. CDKN1A (p21) mRNA expression analysis of knock-out cells during the first 24 h after dTMP withdrawal. G. mRNA expression analysis of pluripotency markers NANOG, OCT4 and SOX2. Results shown as ratio of mRNA over CYCLOG mRNA. H. Immunocytochemistry analysis of pluripotency markers NANOG, OCT4, SOX2 and SSEA4, alone or merged with a nuclear dye channel (Hoechst). I. Karyotype of wild-type hiPSC, passage 20. Karyotype of TYMS-knock-out hiPSC, passage 20 (left) and passage 45 (right). Statistical significance in panels A, F and G based on Wilcoxon test; $p > 0.05$ (ns, not shown), $p < 0.05$ (*), $p < 0.01$ (**), $p < 0.001$ (***).

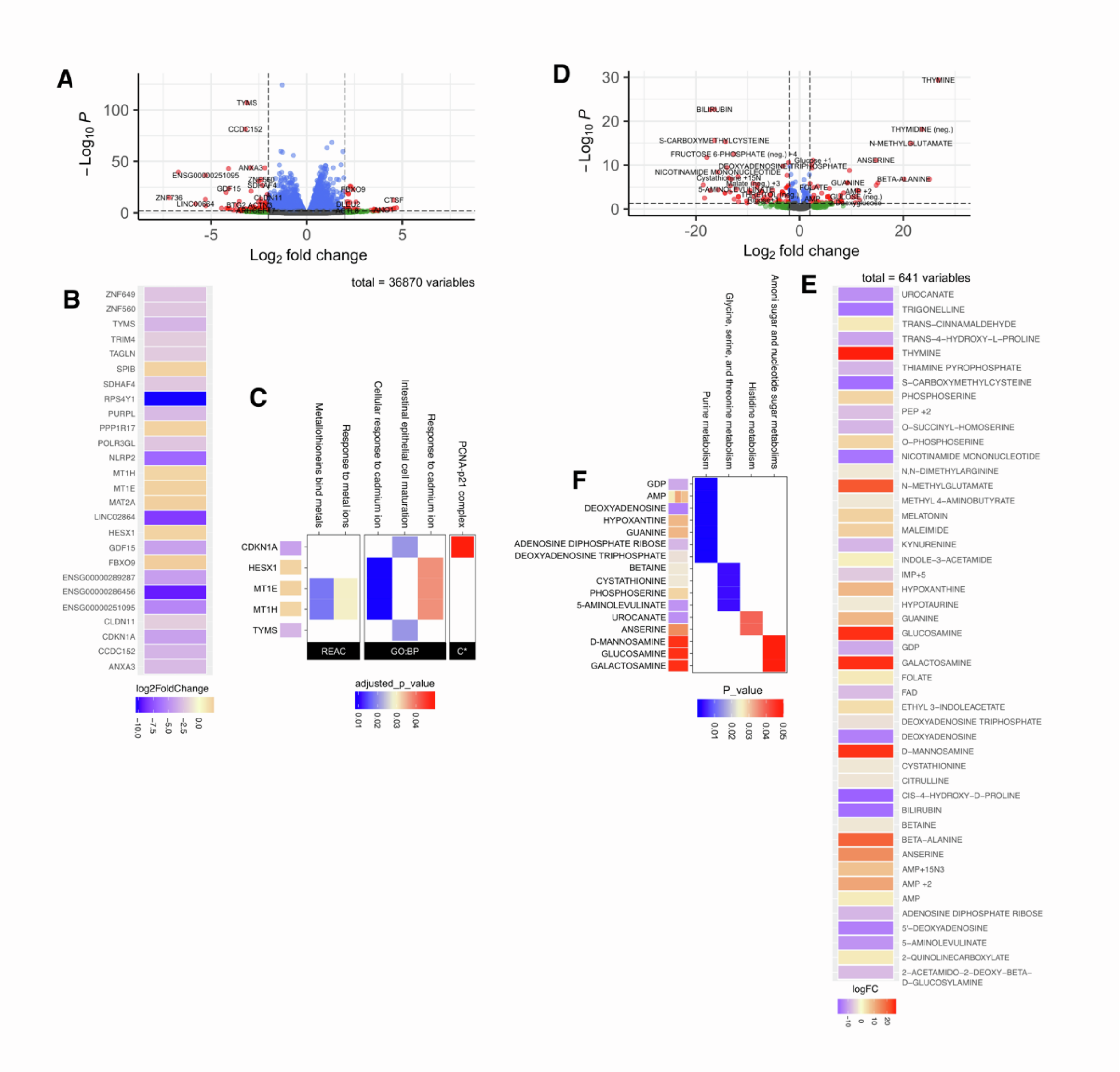

Figure S2. A. Volcano plot depicting significantly changed genes in knock-out hiPSC compared to the wild-type. Fold change threshold: 2; P-value threshold: 0.01. B. Heatmap of significantly changed genes. C. Summary of significantly afected pathways from gProfiler, including genes considered in the analysis. D. Volcano plot depicting significantly changed metabolites in knock-out hiPSC compared to the wild-type. Fold change threshold: 2; P-value threshold: 0.05. E. Heatmap of significantly changed metabolites. F. Summary of significantly affected pathways from Metaboanalyst, including metabolites considered in the analysis.

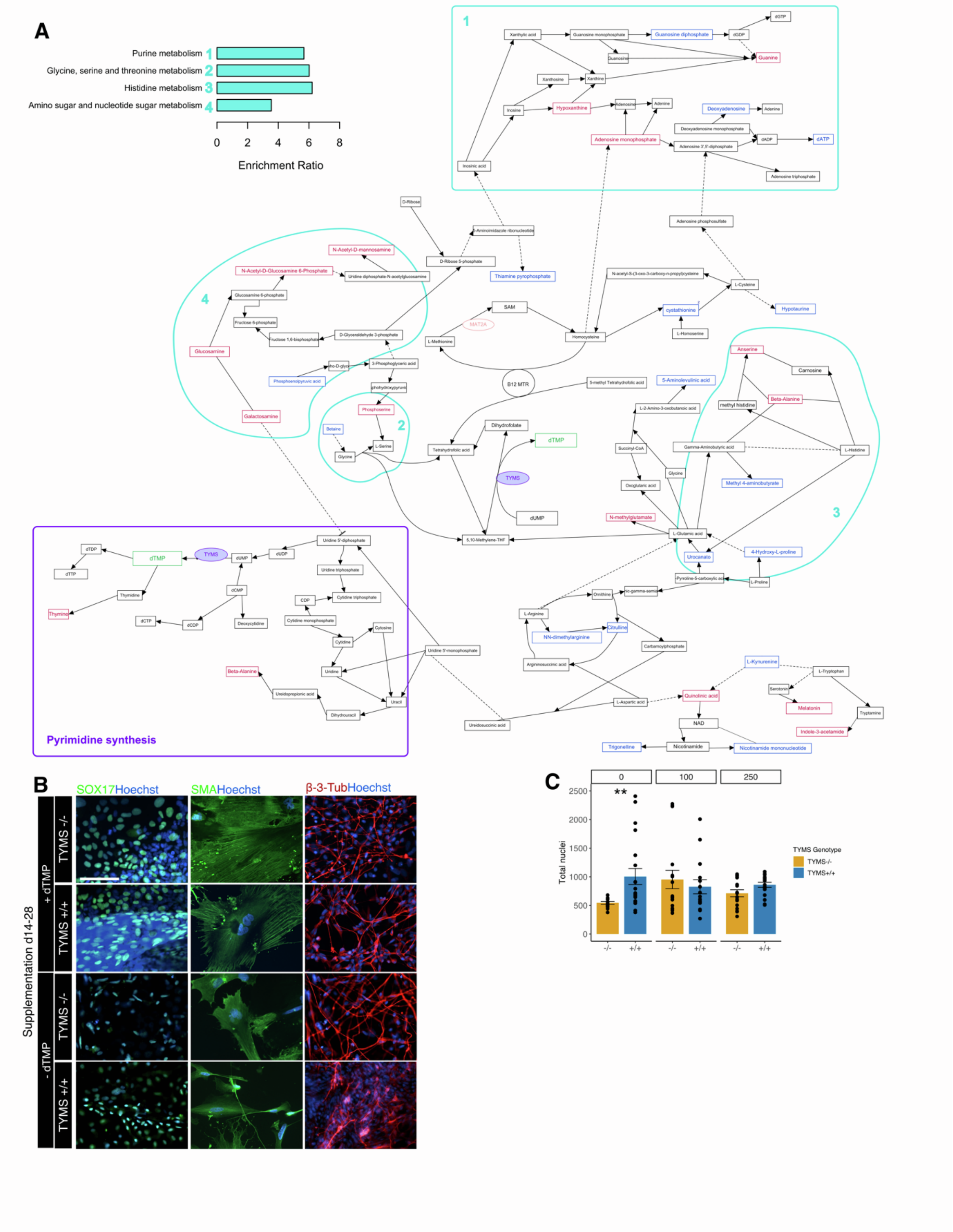

Figure S3. A. Map of metabolic landscape based on the significantly enriched metabolic pathways from metabolomics analysis (circled in cyan). Pyrimidine biosynthesis circled in purple. B. Immunocytochemistry against markers for endoderm (SOX17), mesoderm (SMA), and ectoderm (beta-3-tubulin) in cells derived from wild-type and knock-out hiPSC without

dTMP supplementation during the first stage of differentiation. Scalebar = 100 μM. C. Quantification of total nuclei per image of 6 week teratomas in mice supplemented with 0, 100 or 250 mg/kg/day of dTMP. Statistical significance based on Wilcoxon test; $p > 0.05$ (ns, not shown), $p < 0.01$ (**).

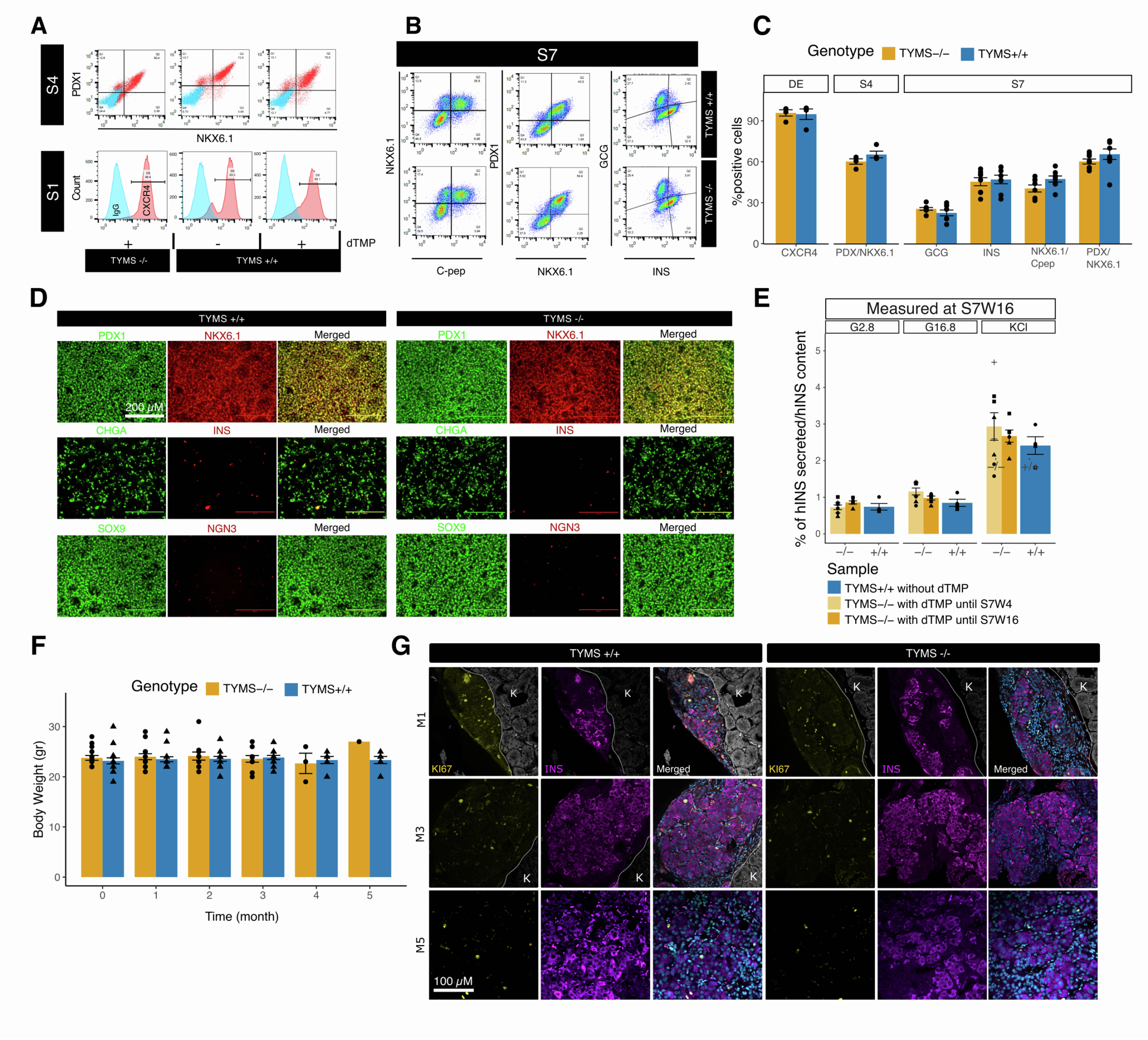

Figure S4. A. Flow cytometry analysis of characteristic markers for definite endoderm (CXCR4) and stage 4 (NKX6.1 and PDX1) of beta-cell differentiation protocol. B. Flow cytometry analysis of markers at stage 7 of beta-cell differentiation: NKX6.1, C-peptide, PDX1, insulin (INS) and glucagon (GCG). C. Flow cytometry analysis for markers for different stages of differentiation: CXCR4 for definite endoderm (DE) stage; NKX6.1 and PDX1 for stage 4 (S4); NKX6.1, C-peptide, PDX1, insulin (INS) and glucagon (GCG) for S7. D. Immunocytochemistry analysis of maturation markers at S4 of beta-cell differentiation protocol. First row: PDX1 and NKX6.1. Second row: CHGA and INS. Third row: SOX9 and NGN3. E. In vitro insulin secretion in response to low (2.8) and high (16.8) glucose concentration and glucose plus KCl (2.8 KCl) at S7W16 (3 months). F.

Expression analysis of mitochondrial genes over human APP. G. Body weight of mice after 1-, 2- and 3-months from implantation (month 0). H. Immunocytochemistry against KI67 (yellow) on insulin positive cells (magenta) from beta-cell grafts at 1, 3 or 5 months after implantation under the kidney capsule (M1, M3, and M5 respectively). K = kidney.

Table S1– gRNAs used for CRISPR/Cas9 TYMS knock-out

Gene	Target	gRNA sequence	PAM	On target	Off target
TYMS	Intron 4	CAACTCATATGGTGGAGACC	AGG	94	54
	Intron 5	TCTGTTAGTGCGTATACCAC	AGG	77	88

Table S2 – PCR and sequencing primer sequences for CRISPR/Cas9 off-targets

gRNA	Location	MM	Primer Sequence (5'-3')	Product length	Algorithm	Assay
1	chr5_54455571	4	AAAGTGCACTGCTGACTGGG	586	CRISPOR	PCR/S
			AGACCACTTCTCAGAGGGGA			
	chr4_24900433	4	CACCCACCATTCTGAGGACC	528		PCR/S
			ATCCGTGTCACCATTCCCAC			
	chr8:-11058099	2	TCCTGACTCCTTCAGTGGGG	404	Benchling	PCR/S
			AAAGATACCACCGCCTCCCA			
	chr17:-43138461	4	GTCGGTCCCAGGTGTTTCTC	587		PCR/S
			ACCACTGGCTTTCAGGCTAC			
	chr19:+7152901	2	CATTCAGACTCCACCCACCC	373		PCR/S
			TCAGCCGCAGAGACTTGAG			
	chr14:+44505493	4	GAGGCTGAAGCTCAAGAGGG	514	iDT	PCR/S
			GCCCCAGCTAAAGTAGAGCC			
2	chr1_233467802	4	AGGCCCTGTAACTCCCTTCT	600	CRISPOR	PCR/S
			CCAGCACCATCACTCCAAGT			
	chr13_26897943	3	TGCACGTTCAGCTTGTGACT	507		PCR/S
			ACAACTCTGCCTCACATGGAG			
	chr1_38172195	4	CCATCCGATTGTAGTAGGCCC	596		PCR/S
			TCCAGCTGGGCAATACTGTG			
	chr2:+13066090	3	TCCCCACCCTATCTACTACCTC	428	Benchling/iDT	PCR/S
			CAAATTATCCTGGGAATAAATGCAC			
	chr18:-31163947	3	AACACCCATGCTGCTGAGAA	639	Benchling	PCR/S
			TGAGCAGTGCCTGGAATCTC			
	chr9:+86563560	4	CATGAGGTGGCTCAGTGGAG	509	Benchling/iDT	PCR/S
			CCATGGCTCCCAATGCAGTA			

Table S3- Number of mice implanted with iPSC for teratoma formation per supplementation condition

Genotype \ dTMP [mg/kg/day]	0	100	250
$TYMS^{+/+}$	4/4	3/4*	5/5
$TYMS^{-/-}$	1/4	3/4*	5/5
*mouse died before teratoma formation			

Table S4– Antibodies used for immunocytochemistry, immunohistochemistry, western blot, and flow cytometry

Epitope	Origin animal	Conjugate	Dilution	Supplier	Assay
Nanog	Rabbit	N/A	1: 500	Cell Signaling; #D73G4	ICC
OCT4	Mouse	N/A	1:500	Santa Cruz; #sc-8628	ICC
Sox2	Rabbit	N/A	1:500	Cell signalling; #D6D9	ICC
SSEA-4	Mouse	N/A	1:500	ThermoFisher; #MA1-023	ICC
Beta-3-tubulin	Rabbit	N/A	1:500	R&D Systems; #MAB1195	ICC
SMA	Mouse	N/A	1:500	Sigma-aldrich; #A2547	ICC
Sox17	Goat	N/A	1:500	R&D Systems; #AF1924	ICC
Ki-67	Rabbit	N/A	1:500	Leica Microsystems #NCL-Ki67p	ICC/IHC
Insulin	Guinea pig	N/A	1:500	Dako ; #A0564	IHC/FC
Glucagon	Mouse	N/A	1:500	Sigma-Aldrich; #G2654	IHC/FC
Syn	Rabbit	N/A	1;250	Novus Biologicals; #NB120-16659	ICC/IHC
SLC18A1	Rabbit	N/A	1;150		ICC/IHC
NGN3	Sheep	N/A	1:500	R&D Systems; #AF3444	ICC/IHC/FC
PDX1	Goat	N/A	1:250	R&D Systems; #AF2419	ICC/IHC/FC
NKX6.1	Mouse	N/A	1:250	DSHB; #F55A10	ICC/IHC/FC
CHGA	Rabbit	N/A	1:500	Dako; #A0564	ICC/IHC/FC
SOX9	Rabbit	N/A	1:500	Millipore; #AB5535	ICC/IHC/FC
TYMS	Rabbit	N/A	1:2500	Proteintech; #15047-1-AP	WB
Guinea pig	Goat	Red 594	1;500		ICC/IHC/FC
Rabbit	Donkey	Red 594	1:500	Thermofisher: #A21207	ICC/IHC/FC
Rabbit	Donkey	Green 488	1:500	Thermofisher: #A21206	ICC/IHC/FC
Mouse	Donkey	Green 488 Red 594	1:500	Thermofisher: #A21202, #A21203	ICC/IHC/FC
Goat	Donkey	Green 488	1:500	Thermofisher; #A11055	ICC
Rabbit	Goat	HRP	1;5000	Cell Signalling; #7074S	WB
B-actin	Mouse	HRP	1;5000	Santa Cruz; #sc-47778	WB
FC = Flow cytometry, ICC = Immunocytochemistry, IHC = Immunohistochemistry, WB = Western blot					

Table S5 – PCR, qPCR and Sanger sequencing primer sequences for TYMS

Gene	Target	Sequence (5’-3’)	Product length	Assay
TYMS	Intron 4	TCAACTCTACCAGGGTGTAG	1336(WT) 871(KO)	PCR/SS
	Intron 5	CCAACCTCAGCATAGCTTTTG		
	Exon 2	CCTCTGCTGACAACCAAACG	95	qPCR
	Exon 3	GAAGACAGCTCTTTAGCATTTG		qPCR
	Exon 4	TCAGGACAGGGAGTTGACCA	117	qPCR
	Exon 5	CAGCGCCATCAGAGGAAGAT		qPCR
SS: Sanger Sequencing				

Table S6 – qPCR primer sequences for pancreatic differentiation markers

Process	Gene	Differentiation stage	Sequence (5’-3’)	Product length
	GAPDH	Housekeeping	GGTCATCCATGACAACTTTGG	84
			CCATCCACAGTCTTCTGGGT	
Pancreatic differentiation	FOXA2	Definite Endoderm (DE)	AAGACCTACAGGCGCAGCT	93
			CATCTTGTTGGGGCTCTGC	
	CHGA	4	AACCGCAGAC CAGAGGACCA	102
			GTCTCAGCCC CGCCGTAGT	
	NGN3	4	GACGACGCGAAGCTCACCAA	98
			TACAAGCTGTGGTCCGCTAT	
	NKX6.1	4	TATTCGTTGGGGATGACAGAG	91
			TGGCCATCTCGGCAGCGTG	
	PDX1	4	AAGTCTACCAAAGCTCACGCG	52
			CGTAGGCGCCGCCTGC	
	GCG	7	GAAGGCGAGATTTCCCAGAAG	113
			CCTGGCGGCAAGATTATCAAG	
	INS	7	CAGAAGCGTGGCATTGTGGA	82
			GCTGCGTCTAGTTGCAGTAG	
	MAFA	7	GCCAGGTGGAGCAGCTGAA	77
			CTTCTCGTATTTCTCCTTGTAC	
	ARX	7	ACAGACGCGCCTCTAGCATA	81
			GCAGGATGTTGAGCTGCGTG	
	SST	7	CCCAGACTCCGTCAGTTTCT	88
			ACAGCAGCTCTGCCAAGAAG	
	UCN3	7	GCCACAAGTTCATGGGGACGTG	120
			GACCGGCATCAGCATCTCTCC	

Table S7- qPCR primer sequences for pluripotency markers

	Gene	Sequence (5’-3’)	Product length
Pluripotency	OCT4	TTGGGCTCGAGAAGGATGTG	91
		TCCTCTCGTTGTGCATAGTCG	
	SOX2	GCCCTGCAGTACAACTCCAT	85
		TGCCCTGCTGCGAGTAGGA	
	NANOG	CTCAGCCTCCAGCAGATGC	94
		TAGATTTCATTCTCTGGTTCTGG	
	Cyclog	TCTTGTCAATGGCCAACAGAG	84
		GCCCATCTAAATGAGGAGTTG	
